# Stress-Responsive Expression, Subcellular Localization and Protein–Protein Interactions of the Rice Metacaspase Family

**DOI:** 10.3390/ijms160716216

**Published:** 2015-07-17

**Authors:** Lei Huang, Huijuan Zhang, Yongbo Hong, Shixia Liu, Dayong Li, Fengming Song

**Affiliations:** National Key Laboratory for Rice Biology, Institute of Biotechnology, Zhejiang University, Hangzhou 310058, China; E-Mails: leihero2008@163.com (L.H.); zhanghj82@zju.edu.cn (H.Z.); yongbohong@126.com (Y.H.); liurui90913@163.com (S.L.); dyli@zju.edu.cn (D.L.)

**Keywords:** rice, metacaspase, expression patterns, abiotic and biotic stress, subcellular localization, protein–protein interaction

## Abstract

Metacaspases, a class of cysteine-dependent proteases like caspases in animals, are important regulators of programmed cell death (PCD) during development and stress responses in plants. The present study was focused on comprehensive analyses of expression patterns of the rice metacaspase (*OsMC*) genes in response to abiotic and biotic stresses and stress-related hormones. Results indicate that members of the OsMC family displayed differential expression patterns in response to abiotic (e.g., drought, salt, cold, and heat) and biotic (e.g., infection by *Magnaporthe oryzae*, *Xanthomonas oryzae* pv. *oryzae* and *Rhizoctonia solani*) stresses and stress-related hormones such as abscisic acid, salicylic acid, jasmonic acid, and 1-amino cyclopropane-1-carboxylic acid (a precursor of ethylene), although the responsiveness to these stresses or hormones varies to some extent. Subcellular localization analyses revealed that OsMC1 was solely localized and OsMC2 was mainly localized in the nucleus. Whereas OsMC3, OsMC4, and OsMC7 were evenly distributed in the cells, OsMC5, OsMC6, and OsMC8 were localized in cytoplasm. OsMC1 interacted with OsLSD1 and OsLSD3 while OsMC3 only interacted with OsLSD1 and that the zinc finger domain in OsMC1 is responsible for the interaction activity. The systematic expression and biochemical analyses of the OsMC family provide valuable information for further functional studies on the biological roles of OsMCs in PCD that is related to abiotic and biotic stress responses.

## 1. Introduction

Programmed cell death (PCD) is an important life process that orchestrates cell suicide and keeps the proper metabolism function [1,2]. During PCD, a kind of enzymes, known as caspases, are often activated and thus initiate the cell death program [3,4,5,6]. In mammals, caspases are a family of cysteine-dependent aspartate-directed proteases, which cleave a variety of intracellular polypeptides and cause the stereotypic morphological and biochemical changes of the cells [7]. However, higher plants do not have close homologues of caspases, but possess a phylogenetically distant family of caspase-like proteins, called metacaspases [8]. Recent genome-wide characterizations in various plant species have revealed that the plant metacaspases are presented as a multigene family. For example, there are nine members in Arabidopsis [9], 8 or 9 members in rice [10,11] and 6 members in grapevine [12]. Phylogenetic analyses indicate that the plant metacaspases can be divided into two categories, type I and type II, according to the protein structure. The type I metacaspases possess a N-terminal extension prodomain ranging from 80 to 120 amino acids in length and two CxxC-type zinc finger domains, whereas type II metacaspases do not have such structures [9]. Both types of metacaspases have a p20 and a p10 subunits in the C-terminal regions, but the linker region between the two subunits in the type I is shorter than the type II metacaspase [11]. Recent biochemical studies demonstrated that two aspartate residues at the p10 subunit in a tobacco type II metacaspase contribute to the substrate-binding pocket [13] and identified a number of physiological substrates for some of the plant metacaspases [14,15].

Although the activity of some plant metacaspases is post-translationally regulated [16,17,18,19,20,21,22,23,24,25], transcriptional regulation of the gene expression is a major mechanism that modulates the activity of metacaspases in plants. A number of studies have indicated that the expression of the metacaspase genes can be regulated by developmental cues [11,12] and induced by a wide range of abiotic and biotic stresses [26,27,28,29,30,31]. For example, Arabidopsis *AtMC1* and *AtMC3* [32], tomato *LeMCA1* [27], pepper *CaMC9* [28], wheat *TaMCA4* [29] and *Nicotiana benthamiana NbMCA1* [30] were previously reported to be upregulated by infection from fungal or bacterial pathogens. Recent functional studies using overexpression or knockout/knockdown approaches have demonstrated that the metacaspases play critical roles in regulating PCD involved in developmental processes and stress responses. Two type II metacaspases, Arabidopsis AtMC9 and Norway spruce mcII-Pa, were found to regulate PCD that are required for the formation of xylem vessel elements [33] and embryogenesis [22,34], respectively. In Arabidopsis, AtMC1 and AtMC3, two type I metacaspases, were shown to positively and negatively regulate PCD, respectively [35], while overexpression of *AtMC8* increased oxidative stress-induced PCD [26]. Knockout of *AtMCP2d*, coding for a type II metacaspase, suppressed PCD induced by fumonisin B1, oxidative stress and *Pseudomonas syringae* [24]. Additionally, the pepper CaMC9 [28], wheat TaMC4 [29] and *N. benthamian* NbMCA1 [30] are positive regulators of PCD and defense response. It was recently found that the functions of Arabidopsis AtMC1 and Norway spruce mcII-Pa are linked to autophagy [36,37].

Subcellular localization and interaction relationships with other proteins are critical to the mode of action for plants’ metacaspases. For example, the Arabidopsis AtMC9 was found to be present in apoplast, nucleus, and cytoplasm and its subcellular localization can be changed during late autolysis process [14,33]. In regulation of PCD, AtMC1 is a positive regulator via interacting with AtLSD1, a negative regulator of cell death [38], whereas AtMC2 is a negative regulator with weak interaction with AtLSD1 [35].

The rice metacaspase (OsMC) family contains eight members [10,11]. Among them, three (OsMC1–3) belong to type I and the remaining five (OsMC4–8) are members of type II [11]. However, very little is known about the biological function of the rice metacaspases so far. As a first step toward understanding the function of rice metacaspases in abiotic and biotic stress-related PCD, we performed a comprehensive analysis of gene expression in response to abiotic and biotic stresses as well as to stress-related hormones. We also analyzed the subcellular localizations of all rice metacaspases and examined the possible interactions of type I with OsLSDs, negative regulators of PCD [38]. Our results presented in this study provide valuable information for further functional studies on the biological roles of rice metacaspases in PCD that is related to abiotic and biotic stress responses.

## 2. Results

### 2.1. Expression Patterns of OsMCs in Response to Abiotic Stresses

Abiotic stresses such as dehydration (e.g., drought and salt) and extreme temperature (e.g., cold and heat) are the main deleterious factors affecting on plant growth/development and ultimately crop yield [39]. To explore the involvement of *OsMCs* in abiotic stresses, the expression patterns of *OsMCs* in rice plants after drought, salt, cold, and heat stresses were analyzed.

#### 2.1.1. Expression Patterns in Response to Drought and Salt Stresses

**Figure 1:**
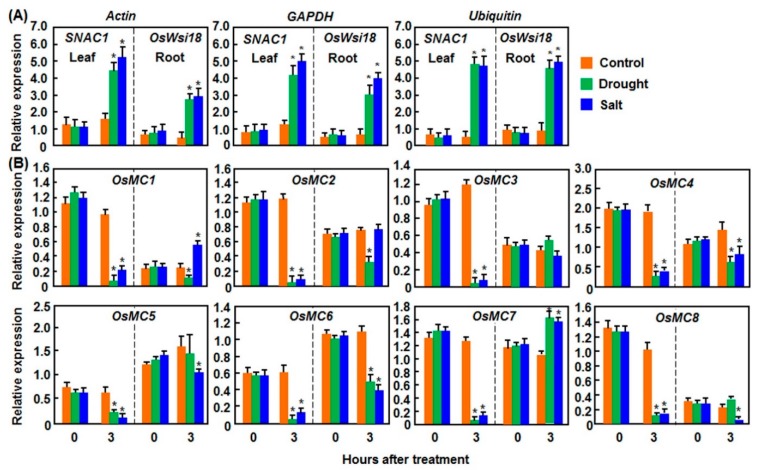
Expression patterns of *OsMCs* in rice leaf and root tissues after treatment with drought and salt stresses. Drought stress was applied to two-week-old seedlings by transferring to three layers of filter papers for fast dehydration. Salt stress was applied to two-week-old seedlings by drenching with 150 mM NaCl solution. Leaf and root samples were collected at indicated time points for qRT-PCR analyses. (**A**) Expression patterns of *SNAC1* and *OsWsi18* calculated with different reference genes; and (**B**) Expression patterns of *OsMCs.* Relative expression is shown as folds of transcript level of different reference genes (**A**) or *Actin* gene (**B**). Left and right parts in each graph divided by dashed lines represent the expression levels in leaf and root tissues, respectively. Data presented are the means ± SD from thee independent experiments and * above the columns indicate significant difference at *p* < 0.05 level.

We first analyzed the expression patterns of *OsMCs* in rice leaves and roots under drought and salt stresses (Figure 1). To assess the accuracy of qRT-PCR [40] and confirm the drought and salt stresses applied to the experimental rice plants, we examined the expression changes of *SNAC1* (*Stress-responsive NAC 1*) in leaves and *Wsi18* (*Water stress-induced 18*) in roots, which are drought- and salt-responsive in leaf and root tissues, respectively [41,42], after drought and salt stresses. As shown in Figure 1A, the expression of *SNAC1* in leaf tissues of plants treated with drought or salt stress was significantly induced while the expression of *OsWsi18* in root tissues of plants treated with drought or salt stress was also upregulated, as compared with corresponding controls in rice plants without stress treatment. Meanwhile, the expression levels of *SNAC1* and *OsWsi18* calculated with three different reference genes such as *Actin*, *GAPDH*, and *Ubiquitin* [43] exhibited similar patterns (Figure 1A). These indicated that the drought and salt treatments in our stress experiments were satisfactory for further analyses of the expression patterns of *OsMCs* in response to drought and salt stresses. In leaf tissues of rice plants treated with drought or salt stress, the expression levels of all *OsMC* genes were significantly downregulated at 3 h after treatment, leading to >3-fold of reduction, as compared with that in control plants without stress treatment (Figure 1B). However, the expressions of *OsMCs* in root tissues of rice plants treated with drought and salt stresses exhibited quite diverse patterns as compared with the patterns in leaf tissues. In drought stress-treated rice plants, only the expression of *OsMC7* was slightly induced, while the expression of *OsMC3*, *OsMC5*, and *OsMC8* was not affected (Figure 1B). By contrast, the expression levels of *OsMC1*, *OsMC2*, *OsMC4*, and *OsMC6* were significantly decreased, leading to >1-fold of reduction, as compared with those in the control plants (Figure 1B). In salt stress-treated rice plants, the expression of *OsMC2* and *OsMC3* was not affected, whereas the expression levels of *OsMC1* and *OsMC7* were increased by approximately 1-fold (Figure 1B). By contrast, the expression levels of *OsMC4*, *OsMC5*, *OsMC6*, and *OsMC8* were significantly decreased, as compared with those in control plants (Figure 1B).

#### 2.1.2. Expression Patterns in Response to Cold and Heat Stresses

**Figure 2:**
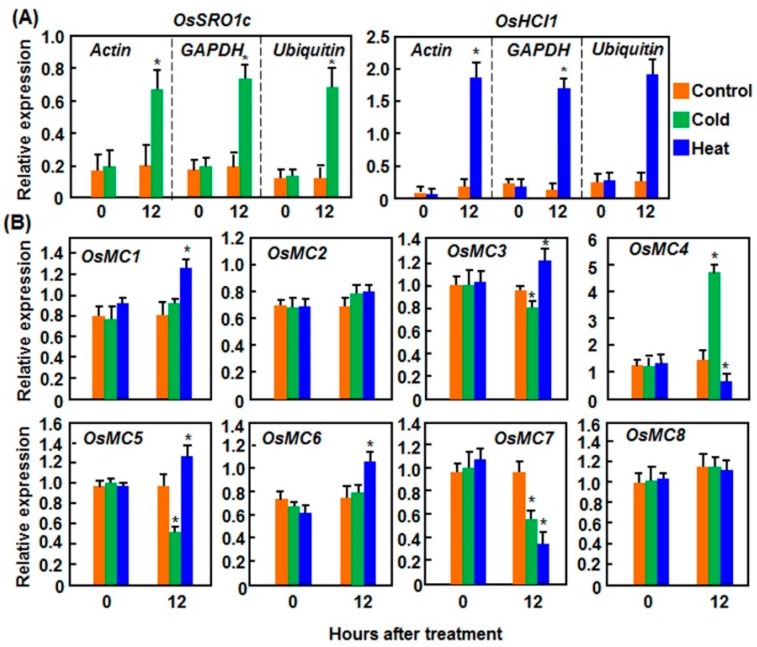
Expression patterns of *OsMCs* in response to cold and heat stresses. Cold and heat stresses were applied by placing two-week-old seedlings in growth chambers with temperatures set at 4 and 42 °C, respectively. Leaf samples were collected at indicated time points for qRT-PCR analyses. (**A**) Expression patterns of *OsSRO1c* and *OsHCI1* calculated with different reference genes; and (**B**) Expression patterns of *OsMCs.* Relative expression is shown as folds of transcript levels of three different reference genes (**A**) or *Actin* gene (**B**). Data presented are the means ± SD from thee independent experiments and * above the columns indicate significant difference at *p* < 0.05 level.

We next analyzed the expression patterns of *OsMCs* in rice leaves under cold and heat stresses (Figure 2). Similarly, we examined the expression changes of *OsSRO1c* (*SIMILAR TO RCD ONE 1c*) and *OsHCI1* (*Heat and cold induced 1*), which were reported to be strongly induced by cold and heat stresses, respectively [44,45], after cold and heat stresses to confirm the stress treatment applied to the experimental rice plants. As shown in Figure 2A, the expression of *OsSRO1c* and *OsHCI1* in leaf tissues of plants treated with cold and heat stresses was significantly induced, respectively, at 12 h after treatment, as compared with corresponding controls without stress treatment. The expression levels of *OsSRO1c* and *OsHCI1* calculated with three different reference genes such as *Actin*, *GAPDH*, and *Ubiquitin* [43] exhibited similar patterns (Figure 2A). The data indicated that the cold and heat treatments were effective to the rice plants in our stress experiments, which were satisfied for analyses of the expression patterns of *OsMCs* in response to cold and heat stresses. In cold stress-treated plants, the expression level of *OsMC4* was significantly induced, resulting in >4-fold of increase at 12 h after treatment, while the expression levels of *OsMC3*, *OsMC5*, and *OsMC7* were decreased, as compared with those in the control plants (Figure 2B). The expression of *OsMC1*, *OsMC2*, *OsMC6*, and *OsMC8* was not affected by cold stress (Figure 2B). In heat stress-treated plants, the expression levels of *OsMC4* and *OsMC7* were significantly decreased, giving >1-fold of reduction; whereas the expression levels of *OsMC1*, *OsMC3*, *OsMC5*, and *OsMC6* were slightly induced (Figure 2B). By contrast, the expression of *OsMC2* and *OsMC8* was not affected by heat stress (Figure 2B).

Collectively, these data indicate that the expression of *OsMCs* display differential expression patterns upon different abiotic stresses and such differential expression may imply their involvement in different networks and have different biological functions.

### 2.2. Expression Patterns of OsMCs in Response to Pathogens

To explore the possible involvement of *OsMCs* in response to biotic stress, we analyzed the expression patterns of the *OsMCs* genes in rice after infection with different types of pathogens. Rice blast disease caused by *Magnaporthe oryzae* and sheath blight disease caused by *Rhizoctonia solani* are two of the most important fungal diseases of rice, whereas the bacterial blight disease caused by *Xanthomonas oryzae* pv. *oryzae* (*Xoo*) is a major rice bacterial disease. Among the thee diseases chosen, *M. oryzae* and *Xoo* are generally (hemi)biotrophoic pathogens while *R. solani* is a typical necrotrophic fungus.

#### 2.2.1. Expression Patterns in Response to *M. oryzae*

**Figure 3:**
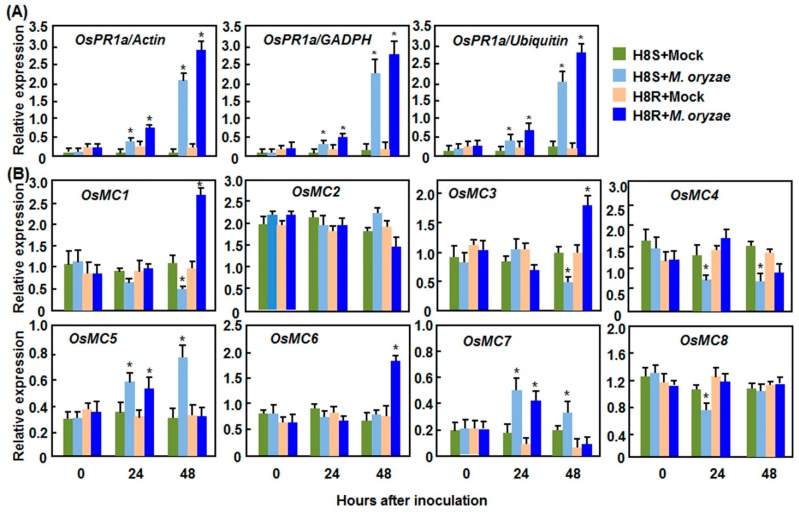
Expression patterns of *OsMCs* in incompatible and compatible interactions between rice and *Magnaporthe oryzae*. Two-week-old seedlings were inoculated by spraying with spore suspension of *M. oryzae* and leaf samples were collected at indicated time points after inoculation for qRT-PCR analyses. (**A**) Expression patterns of *OsPR1a* calculated with different reference genes; and (**B**) Expression patterns of *OsMCs.* Relative expression is shown as folds of transcript levels of three different reference genes (**A**) or *Actin* gene (**B**). Data presented are the means ± SD from thee independent experiments and * above the columns indicate significant difference at *p* < 0.05 level.

In rice-*M. grisea* interactions, a pair of near isogenic lines (NILs) H8R and H8S interact differentially with strain 85-14-B1 of *M. oryzae*, resulting in incompatible and compatible interactions, respectively [46]. We first confirmed the induction of defense response in incompatible and compatible interactions between rice and *M. oryzae* by analyzing the expression changes of known defense-related genes in rice plants after inoculation. As shown in Figure 3A, the expression levels of *OsPR1a* (*Pathogenesis-related 1a*), a well-known defense-related gene that is induced by *M. oryzae* [47], in both of the incompatible and compatible interactions were significantly increased at 48 h after inoculation. Meanwhile, typical disease symptom appeared in leaves of H8S plants while small restricted disease spots was observed on leaves of H8R plants at 3 days after inoculation. Similar expression patterns of *OsPR1a* were observed when calculated with three different reference genes such as *Actin*, *GAPDH*, and *Ubiquitin* [43] (Figure 3A). These data demonstrate the effectiveness and reliability of our pathogen inoculation experiments. As compared with those in the mock-inoculated plants, the expression levels of *OsMC1*, *OsMC3*, and *OsMC6* were significantly induced in leaves of H8R plants at 48 h after inoculation, leading to 2~3-fold of increases, while the expression levels of *OsMC1* and *OsMC3* in H8S plants was decreased (Figure 3B). Similar expression patterns and dynamics were observed for *OsMC5* and *OsMC7* (Figure 3B). The expression of both *OsMC5* and *OsMC7* in incompatible and compatible interactions was induced at 24 h after inoculation; however, increased expression at 48 h after inoculation was only observed in compatible interaction (Figure 3B). Additionally, the expression of *OsMC4* and *OsMC8* was decreased only in compatible interaction (Figure 3B). The expression of *OsMC2* was not affected by infection of *M. grisea* in both of the incompatible and compatible interactions (Figure 3B).

#### 2.2.2. Expression Patterns in Response to *Xoo*

**Figure 4:**
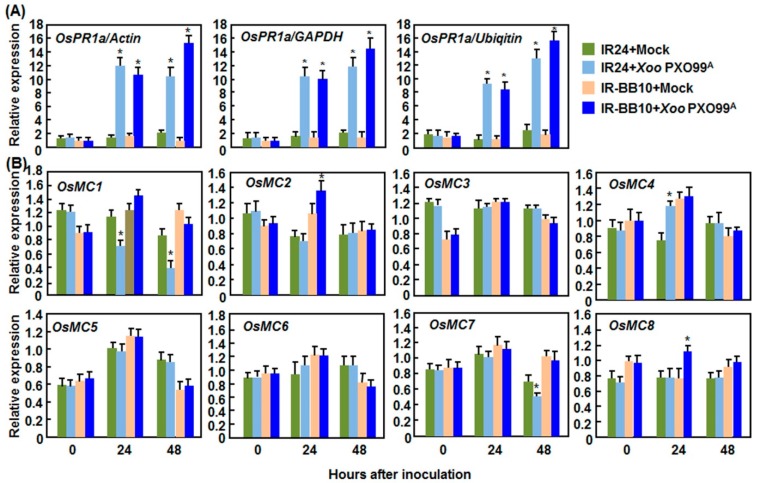
Expression patterns of *OsMCs* in the incompatible and compatible interactions between rice and *Xanthomonas oryzae* pv. *oryzae*. Eight-week-old seedlings were inoculated with *X. oryzae* pv. *oryzae* strain PXO99^A^ by leaf clipping method and leaf samples were collected at indicated time points after inoculation for qRT-PCR analyses. (**A**) Expression patterns of *OsPR1a* calculated with different reference genes; and (**B**) Expression patterns of *OsMCs.* Relative expression is shown as folds of transcript levels of three different reference genes (**A**) or *Actin* gene (**B**). Data presented are the means ± SD from thee independent experiments and * above the columns indicate significant difference at *p* < 0.05 level.

In rice-*Xoo* interactions, a pair of NILs IR-BB10 and IR24 interact differentially with *Xoo* strain PXO99^A^, resulting in incompatible and compatible interactions, respectively [48]. Also, we examined the expression changes of defense-related genes as a sign of activation of defense response in incompatible and compatible interactions between rice and *Xoo* before analyzing the expression patterns of *OsMCs*. As shown in Figure 4A, the expression levels of *OsPR1a*, a defense-related gene that is induced by *Xoo* [49], both the incompatible and compatible interactions were significantly increased at 48 h after inoculation. Meanwhile, typical disease symptoms appeared in the inoculated leaves of IR-BB10 and IR24 plant at 7 days after inoculation, but the IR-BB10 plants showed a disease resistance phenotype with shorter lesions than the IR24 plants. Similar expression patterns of *OsPR1a* were observed when calculated with three different reference genes such as *Actin*, *GAPDH*, and *Ubiquitin* [43] (Figure 4A). These data demonstrate the effectiveness and reliability of our pathogen inoculation experiments. Surprisingly, the expression of most of the *OsMC* genes was not affected significantly in both of the IR-BB10 and IR24 plants after *Xoo* infection (Figure 4B). However, the expression levels of *OsMC1* at 24 and 48 h and *OsMC7* at 48 h were significantly decreased while the expression level of *OsMC4* at 24 h was induced markedly in IR24 plants after *Xoo* infection, but the levels in IR-BB10 plants were not affected (Figure 4B). By contrast, the expression of *OsMC2* and *OsMC8* in IR-BB10 plants was induced significantly at 24 h after *Xoo* infection (Figure 4B).

#### 2.2.3. Expression Patterns in Response to *R. solani*

**Figure 5:**
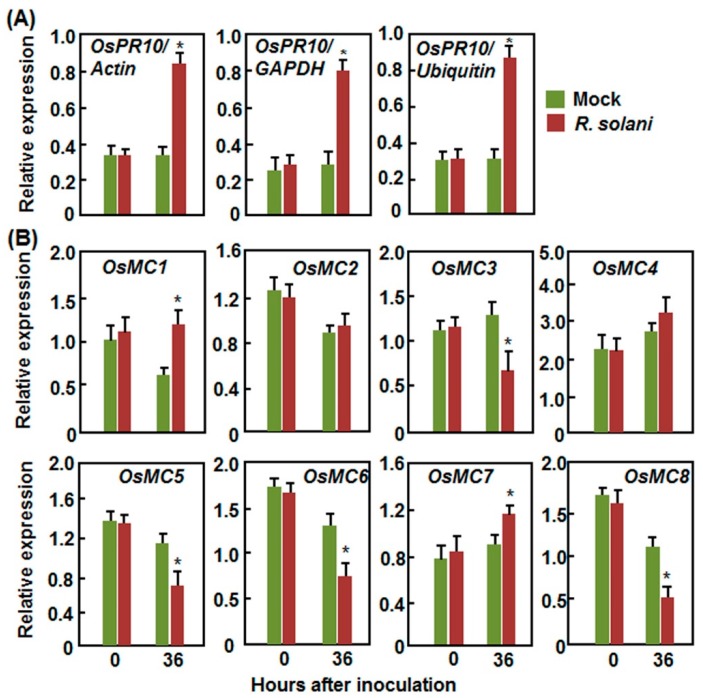
Expression patterns of *OsMCs* in response to *Rhizoctonia solani*. Six-week-old plants were inoculated by attaching mycelial of *R. solani* strain GD-118 onto sheath and leaf samples were collected at indicated time points after inoculation for qRT-PCR analyses. (**A**) Expression patterns of *OsPR10* calculated with different reference genes; and (**B**) Expression patterns of *OsMCs.* Relative expression is shown as folds of transcript levels of three different reference genes (**A**) or *Actin* gene (**B**). Data presented are the means ± SD from three independent experiments and * above the columns indicate significant difference at *p* < 0.05 level.

In rice-*R. solani* interaction, the expression level of *OsPR10* (*Pathogenesis-related 10*), a defense-related gene that is induced by *R. solani* [50], was significantly increased by 2 folds at 36 h after inoculation, as compared with that in the mock-inoculated plants (Figure 5A). Similar expression patterns of *OsPR10* were observed when calculated with three different reference genes such as *Actin*, *GAPDH*, and *Ubiquitin* [43] (Figure 5A). Therefore, the effectiveness and reliability of our pathogen inoculation experiments were satisfied for further analyses of expression of *OsMCs* in response to *R. solani*. Among the *OsMCs*, the expression of *OsMC1* and *OsMC7* was induced significantly by *R. solani* as compared with that in the mock-inoculation plants (Figure 5B). By contrast, the expression of *OsMC3*, *OsMC5*, *OsMC6*, and *OsMC8* was downregulated by *R. solani* while the expression of *OsMC2* and *OsMC4* was not affected by *R. solani* (Figure 5B).

Together, these data indicate that the expression of *OsMCs* exhibit differential expression patterns in rice upon infections from different fungal and bacterial pathogens and such differential expression may imply their involvement in defense response to pathogens.

### 2.3. Expression Patterns of OsMCs in Response to Stress-Related Hormones

**Figure 6:**
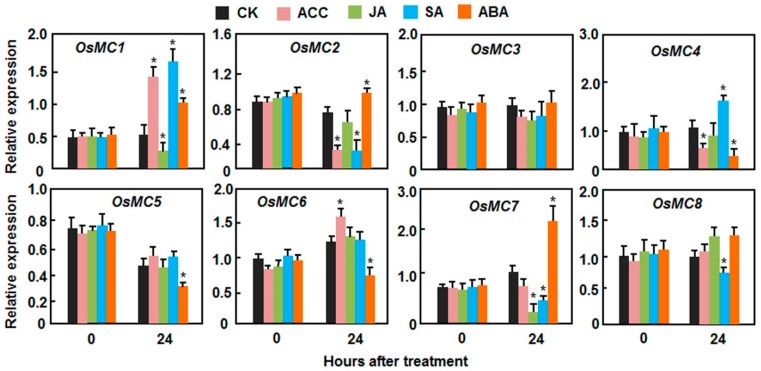
Expression patterns of *OsMCs* in response to stress-related hormones. Two-week-old seedlings were sprayed with 100 µM SA, 100 µM MeJA, 100 µM ACC, 100 µM ABA or similar volumes of solution as controls. Leaf samples were collected at indicated time points and relative expression of the genes is shown as folds of transcript level of the actin gene. Data presented are the means ± SD from thee independent experiments and * above the columns indicate significant difference at *p* < 0.05 level.

Abscisic acid (ABA), salicylic acid (SA), jasmonic acid (JA), and ethylene (ET) are well-known stress-related hormones in plants and are involved in abiotic and biotic stress responses [51,52,53,54]. The differential expression patterns of *OsMCs* in response to multiple abiotic and biotic stresses led us to examine the responsiveness of *OsMCs* to these stress-related hormones. As shown in Figure 6, the *OsMC* genes responded differentially as revealed by their expression changes in plants after treatment with ABA, SA, JA, or 1-amino cyclopropane-1-carboxylic acid (ACC, a precursor of ET). In ACC-treated plants, dramatic expression changes of *OsMC1*, *OsMC2*, *OsMC4* and *OsMC6* were observed (Figure 6). The expression levels of *OsMC1* and *OsMC6* were increased while the expression levels of *OsMC2* and *OsMC4* were decreased at 24 h after treatment (Figure 6). The expression of *OsMC3*, *OsMC5*, *OsMC7*, and *OsMC8* was not affected by ACC treatment (Figure 6). After JA treatment, the expression levels of *OsMC1* and *OsMC7* were significantly decreased while the expression of *OsMC2*, *OsMC3*, *OsMC4*, *OsMC5*, *OsMC6*, and *OsMC8* was not affected (Figure 6). In response to SA treatment, the expression levels of *OsMC1* and *OsMC4* were upregulated by 3- and 1.5-fold, respectively, whereas the expression levels of *OsMC2*, *OsMC7*, and *OsMC8* were downregulated significantly by >1.5-fold (Figure 6). The expression of *OsMC3*, *OsMC5*, and *OsMC6* was not affected by SA treatment (Figure 6). After ABA treatment, the expression levels of *OsMC1*, *OsMC2*, and *OsMC7* were increased while the expression levels of *OsMC4*, *OsMC5*, and *OsMC6* were decreased (Figure 6). The expression of *OsMC3* and *OsMC8* was not affected by ABA treatment (Figure 6). Taken together, the *OsMC* genes respond to multiple stress-related hormones with differential expression patterns, which may imply their relationships with the stress hormone-mediated signaling for their biological functions.

### 2.4. Putative Cis-Elements in Promoter Regions of the OsMC Genes

To gain insights into the transcriptional regulation of the *OsMC* genes, putative *cis*-elements in the promoter regions were examined. A number of well-known *cis*-elements that are involved in growth/development and stress response were detected in the 1 kb upstream regions of the *OsMC* genes (Table 1). One MYB-related (S000176), one MYC-related (S000407) and one WRKY-related (S000447) *cis*-elements, which are known to be involved in abiotic and biotic stress responses [55,56], were found in the promoter regions of all *OsMC* genes. Two other WRKY-related (S000390 and S000457) *cis*-elements were also detected in the promoter regions of the *OsMC* genes except the *OsMC6* (Table 1). The promoter regions of *OsMC3*, *OsMC4*, *OsMC5*, *OsMC6*, and *OsMC8* further contain GCC-box (S000430), known recognition site for ERFs [57]. Three ABA-responsive *cis*-elements, ABRELATERD1 (S000414), ABREOSRAB21 (S000012), and ABRERATCAL (S000408), were found in the promoter regions of *OsMC2*, *OsMC3*, *OsMC6*, and *OsMC8*; however, the promoter regions of *OsMC1*, *OsMC4*, *OsMC5*, and *OsMC7* do not contain known ABA-responsive *cis*-element (Table 1). Additionally, both of the *OsMC1* and *OsMC5* promoter regions harbor one NtBBF1motif (S000273), which is known to be auxin-responsive [58].

**Table 1:** Putative *cis*-elements in promoter regions of the *OsMC* genes.

Regulator	*Cis*-Element	Code	Numbers of *cis*-Elements							
			*OsMC1*	*OsMC2*	*OsMC3*	*OsMC4*	*OsMC5*	*OsMC6*	*OsMC7*	*OsMC8*
ABA	ABRELATERD1	S000414	–	2	1	–	–	2	–	1
	ABREOSRAB21	S000012	–	–	2	–	–	–	–	–
	ABRERATCAL	S000408	–	3	–	–	–	2	–	1
MYB	MYBCORE	S000176	2	4	5	1	1	2	1	1
MYC	MYCCONSENSUSAT	S000407	4	14	8	4	4	8	6	8
WRKY	WBOXATNPR1	S000390	1	1	1	–	4	–	–	3
	WBOXNTERF3	S000457	2	1	1	1	2	–	1	6
	WRKY710S	S000447	3	4	3	4	6	1	1	10
ERF	GCCCORE	S000430	–	–	3	1	2	2	–	1
Auxin	NTBBF1ARROLB	S000273	1	–	–	–	1	–	–	–

### 2.5. Subcellular Localization of OsMCs

**Figure 7:**
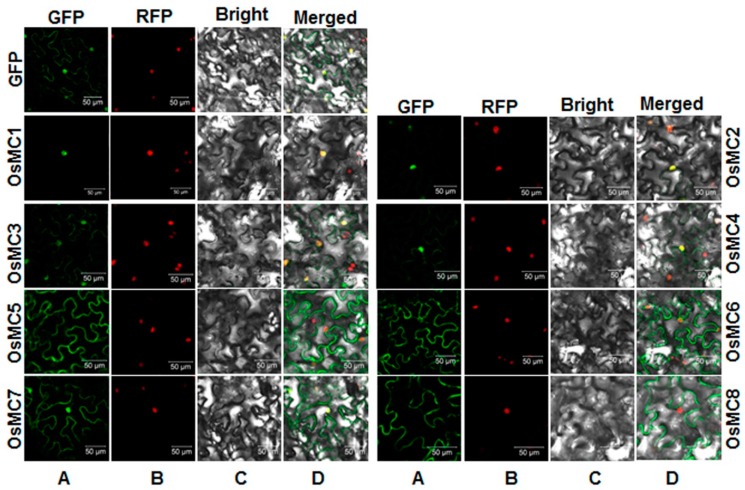
Subcellular localization of the OsMC proteins when transiently expressed in leaves of *Nicotiana benthamiana*. Agrobacteria harboring pFGC-OsMC1/2/3/4/5/6/7/8 or pFGC-Egfp were infiltrated into leaves of *N. benthamiana* plants expressing a red nucleus marker protein RFP-H2B and leaf samples were collected at 24 h after agroinfiltration. Images were observed and taken under a confocal laser scanning microscope in dark field for green fluorescence (**A**); red fluorescence (**B**); white field for cell morphology (**C**) and in combination (**D**), respectively. Bar = 50 µM.

To explore the subcellular localization of the OsMC proteins, we first searched putative nuclear localization signal (NLS) in the OsMC proteins by PSORT analysis. Putative NLSs were identified in OsMC1 and OsMC7, which contain NLS of RRRH and PVKGRRH sequences, respectively. No NLS was detected in OsMC2, OsMC3, OsMC4, OsMC5, OsMC6, and OsMC8 proteins. We then examined experimentally the subcellular localization of OsMC proteins though transient expression of GFP:OsMC fusions in leaves of *Nicotiana benthamiana* plants that expressed a red nuclear marker RFP–H2B protein [59]. Confocal laser scanning microscope observation at 48 h after agroinfiltration revealed that the GFP:OsMC1 fusion was solely localized to the nucleus, co-localized with the known nucleus marker RFP–H2B protein and the GFP:OsMC2 fusion was mainly localized in the nucleus with a very weak signal in the cytoplasm (Figure 7). The GFP:OsMC3, GFP:OsMC4 and GFP:OsMC7 fusions were found to distribute both in the cytoplasm and nucleus whereas the *GFP:OsMC5*, *GFP:OsMC6*, and GFP:OsMC8 fusions were exclusively localized in the cytoplasm without any localization in the nucleus (Figure 7). The GFP alone was detected in both the cytoplasm and nucleus. These results indicate that the OsMC proteins exhibit differential subcellular localization, which may be associated with their specific biological functions in the cells.

### 2.6. Interaction Relationships between Type I OsMCs and OsLSDs

**Figure 8:**
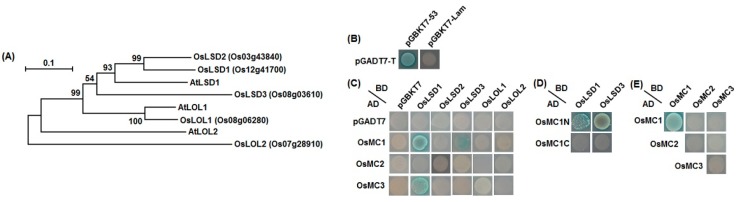
Interactions between type I OsMCs and OsLSDs. (**A**) Phylogenetic tree of rice OsLSDs/OsLOLs with Arabidopsis AtLSD1/AtLOLs. Phylogenetic tree was constructed by Neighbor-joining method using MEGA program; The bar represents 0.1 amino acid substitution per site; (**B**) Positive (pGADT7-T + pGBKT7-53) and negative (pGADT7-T + pGBKT7-Lam) controls; (**C**) Interactions of OsMC1, OsMC2 and OsMC3 with OsLSD1 and OsLSD3 but not with OsLSD2, OsLOL1 and OsLOL2; (**D**) Interactions of N- and C-terminals of OsMC1 with OsLSD1 and OsLSD3; and (**E**) Interactions among OsMC1, OsMC2 and OsMC3. Yeasts harboring the indicated plasmid combinations were grown on selective medium SD/Trp^−^Leu^−^His^−^Ade^−^ and β-galactosidase activity showing positive interactions was examined by addition of X-α-gal. Repeated experiments showed similar results. AD, pGADT7 vector; BD, pGBKT7 vector.

It was previously reported that the Arabidopsis type I metacaspase AtMC1 interacted with AtLSD1 [35], a negative regulator of cell death [38]. To examine whether thee rice type I metacaspases, OsMC1, OsMC2, and OsMC3, can also interact with OsLSDs. In Arabidopsis, there are one AtLSD1 and two LSD One Like proteins (AtLOL1 and AtLOL2) [38,60]. Five putative AtLSD1 homologs were identified in rice genome [10,61] and the nomenclature for these proteins are somewhat confused in literatures. To avoid confusion in nomenclature for these genes, we generated a phylogenetic tree including the rice OsLSDs and OsLOLs and Arabidopsis AtLSD1 and AtLOLs and assigned unique names to them (Figure 8A). OsLOL1 (OsLSD1) and OsLOL2 were previously reported and found to function in PCD, disease resistance and growth [62,63,64]. In our yeast two hybrid experiments, a positive control (pGADT7-T + pGBKT7-53) and a negative control (pGADT7-T + pGBKT7-Lam) were always included to rule out possible false interactions (Figure 8B). As shown in Figure 8C, significant interactions between OsMC1 and OsLSD1/OsLSD3 and between OsMC3 and OsLSD1 were detected; no interaction in other pairwise combinations between OsMC1/2/3 and OsLSDs/OsLOLs was observed. Notably, OsMC2 did not interact with any of OsLSDs while OsLSD2 did not interact with all three type I metacaspases OsMC1, OsMC2, and OsMC3 (Figure 8C). We further examined which region of OsMC1 is responsible for the interaction activity with OsLSD1 and OsLSD3. As shown in Figure 8D, the N-terminal region (1-84 aa), which contains the conserved zinc finger domain, did interact with both of OsLSD1 and OsLSD3, but the C-terminal region of OsMC1 (85–368 aa), which lacked the conserved zinc finger domain, did not interact with OsLSD1 and OsLSD3, indicating that the zinc finger domain in OsMC1 is critical for its interaction activity with OsLSDs. Furthermore, we also examined whether these type I OsMCs interact with each other or itself. Self-interaction in OsMC1 was observed (Figure 8E). However, OsMC1, OsMC2, and OsMC3 did not interact to each other and OsMC2 and OsMC3 did not show self-interaction in yeast (Figure 8E).

## 3. Discussion

Although the rice OsMC family has been previously characterized [10,11], none of the family members has been studied for the biological function so far. The results presented in this study indicate that members of the OsMC family respond differentially to multiple abiotic and biotic stresses as well as to some well-known stress-related hormones. However, the responsiveness of the members of the OsMC family to these stresses or hormones varies to some extent. For example, *OsMC1* was found to be induced by all of the stresses or hormones tested; while other *OsMCs* responded to less stresses or hormones (Figure 1, Figure 2, Figure 3, Figure 4, Figure 5 and Figure 6). Such differential stress- or hormone-related responsiveness may imply the specific involvement of the members of the *OsMC* family in different abiotic and biotic stress responses.

It was previously reported that the Arabidopsis *AtMC1* and *AtMC3*, tomato *LeMCA1*, pepper *CaMC9*, wheat *TaMCA4* and *N. benthamiana NbMCA1* were significantly induced by infection from fungal or bacterial pathogens [27,28,29,30,32], indicating the importance of transcriptional regulation of metacaspase genes for the biological functions in plants. In the present study, we also found that infection with *M. oryzae*, *Xoo*, or *R. solani*, thee different pathogens with distinct infection styles, did cause transcriptional reprogramming in expression of the *OsMC* genes. For example, more than half of the *OsMCs* displayed differential expression in either compatible or incompatible interaction between rice and *M. oryzae*. Among these *OsMCs*, *OsMC5*, a type II metacaspase, was induced by the blast fungus in both compatible and incompatible interactions (Figure 3) but not by SA and JA (Figure 6), two well-known signaling molecules in defense responses. This is in accordance with a previous observation from RNA-Seq analysis of rice samples infected with *M. grisea* [65]. On the other hand, some of the *OsMCs* exhibited differential expression patterns in different rice-pathogen interactions. For example, *OsMC1* was found to be induced by the blast fungus in the incompatible interaction of rice-*M. oryzae* (Figure 3) and by SA (Figure 6); however, its expression was downregulated in the incompatible interaction of rice-*Xoo* (Figure 4). Further, *OsMC7* showed a higher level of expression in compatible rice-*M. oryzae* interaction (Figure 3) but was downregulated by SA and JA (Figure 6), whereas its expression was not affected dramatically in interactions of rice-*Xoo* and rice-*R. solani* (Figure 4 and Figure 5). The differential expression of *OsMC1* and *OsMC7* in response to different pathogens may be attributed to different mechanisms that are used by *M. grisea*, *Xoo*, and *R. solani* during their infection processes [66]. This is similar to the observations that *OsEDR1* was found to play opposite roles in the rice-*M. oryzae* and rice-*Xoo* interactions [67].

Diverse abiotic stresses have been found to induce PCD and the abiotic stress-induced PCD significantly affects plant growth and development [68]. It was found that abiotic stress-induced PCD is often accompanied with accumulation of reactive oxygen species (ROS) [68]. In fact, ROS and other abiotic factors such as ultraviolet light and ozone were reported to induce expression of metacaspase genes in Arabidopsis and maize [26,31]. In the present study, we found that, like the responsiveness to pathogens, members of the *OsMC* family also respond differentially to dehydration (drought and salt) and extreme temperature (cold and heat) stresses. Interestingly, expression of most *OsMC* genes was downregulated by drought and salt stresses (Figure 1 and Figure 2). Some of the *OsMC* genes exhibited overlapping expression patterns under different abiotic stress conditions, indicating their possible involvement in diverse abiotic stress responses. For example, the expression of the *OsMC* genes in leaf tissues was significantly downregulated by drought and salt stresses (Figure 1). However, the downregulated expression patterns of *OsMCs* in leaf tissues are not well correlated with the patterns in the root tissues (Figure 1). This is in agreement with the observations that some of the transcriptional factor genes exhibited distinct expression patterns in leaf and root tissues under abiotic stress conditions [69,70]. Furthermore, the drought and salt stress-caused differential expression patterns of some *OsMCs* are correlated with the patterns in rice plants treated with ABA (Figure 1, Figure 2 and Figure 6), which is believed to play critical roles in response to drought and salt stresses [51,52]. For example, the expression of *OsMC4* was suppressed by drought and salt stresses (Figure 1) and also was suppressed by ABA (Figure 6). On the other hand, opposite expression patterns of *OsMCs* under abiotic stress conditions were also observed. For example, the expression of *OsMC4* was induced by cold stress but suppressed by heat stress, whereas the expression of *OsMC5* was suppressed by cold stress but induced by heat stress (Figure 2), indicating opposite roles for *OsMC4* and *OsMC5* in extreme temperature stresses.

Notably, the range of changes in the expression of *OsMCs* in response to abiotic and biotic stress varied greatly. For example, expression of *OsMC1*, *OsMC3*, and *OsMC6* was induced by 2–3-fold in leaves inoculated with an incompatible strain of *M. oryzae* (Figure 3), whereas the expression changes of some *OsMCs* in response to pathogens or abiotic stress were less than 2-fold (Figure 1, Figure 2, Figure 3, Figure 4and Figure 5). Similar results were also observed for the expression changes of *MC* genes in other plants; for example, 3–4-fold induction for *CamC9* in pepper leaves inoculated with *Xanthomonas campestris* pv. *vesicatoria* [28], ~5-fold upregulation for *TaMC4* in wheat leaves challenged with the avirulent race of *Puccinia striiformis* f. sp. *tritici* [29], ~1.5-fold induction for *NbMCA1* in *N. benthamiana* leaves infected with *Colletotrichum destructivum* [30] and ~1-fold change for *TaeMCAII* in wheat under heat stress [71]. The difference in the expression changes among the *OsMC* genes in response to different pathogens may be explained by the possibility that both of the transcriptional and post-transcriptional regulations are required for the functions of *OsMCs* in given abiotic ad biotic stress responses [16,17].

Several features on the subcellular localization and protein-protein interactions provide valuable information on the mode of action of OsMCs. In the present study, we examined the subcellular localization of all members in the OsMC family and our results indicate that the members of this family exhibit diverse subcellular localization. The OsMC1 protein contains a predicted NLS with sequence of RRRH and was found to be localized in nucleus (Figure 7). This is further supported by the interaction between OsMC1 and OsLSD1 (Figure 8), whose homolog in *Pisum sativa*, PsLSD1, was shown to be a nucleus-localized protein [72]. Further experiments are required to examine the *in vivo* OsMC1-OsLSD1 interaction and whether such interaction takes place in the nucleus. OsMC7, along with OsMC3 and OsMC4, were evenly distributed in the cells (Figure 7), although OsMC7 contains a putative NLS sequence. Interestingly, OsMC5, OsMC6, and OsMC8 were found to be exclusively localized in the cytoplasm without any localization in the nucleus (Figure 7). However, it was previously reported that the Arabidopsis AtMC9 is present in apoplast, nucleus and cytoplasm [14] and its subcellular localization can be changed from an even cytoplasmic localization in living cells to patches or aggregates of different sizes in cells during late autolysis [33]. Thus, whether dynamics in subcellular localization upon developmental and stress signals are the case for the OsMC proteins needs to be examined further. On the other hand, the type I metacaspase, OsMC1 interacted with OsLSD1 and OsLSD3 while OsMC3 only interacted with OsLSD1 (Figure 8). The N-terminal LSD1-type zinc finger domain in OsMC1 is responsible for the interaction activity with OsLSD1 and OsLSD3 (Figure 8). This is in agreement with the interactions between AtMC1 and AtLSD1 [35]. Furthermore, OsMC1 was found to interact with itself while *OsMC2 and OsMC3* did not (Figure 8), indicating possible different modes of action for these type I metacaspases.

## 4. Materials and Methods

### 4.1. Plant Materials and Growth

Rice (*Oryza sativa* L.) cv. Yuanfengzao and two NIL pairs (provided by Dr. Zuhua He, Shanghai Institute of plant physiology and ecology, Chinese Academy of Sciences, Shanghai, China) were used in this study for different purposes. The cv. Yuanfengzao was used for analyses of gene expression in response to abiotic stress, hormone treatments, and infection by *R. solani*. The pair of NILs H8S and H8R was used for analysis of gene expression in compatible and incompatible interactions between rice and *M. oryzae* race ZB1 (provided by Mr. Rongyao Chai, Zhejiang Academy of Agricultural Sciences, Hangzhou, China) while the pair of NILs IR-BB10 and IR24 (provided by International Rice Research Institute, Los Banos, the Philippines) was used for analysis of gene expression in compatible and incompatible interactions between rice and *X. oryzae* pv. *oryzae* (*Xoo*) strain PXO99^A^ (provided by Dr. Jean Leach, Colorado State University, Fort Collins, CO, USA). The rice plants were grown in a growth room under a 14-h light/10-h dark cycle at 26 °C.

### 4.2. Treatments with Abiotic Stress and Hormones

Two-week-old cv. Yuanfengzao seedlings were treated with varied abiotic stresses or hormones. Drought stress was applied by transferring the hydroponically cultivated seedlings to thee layered filter papers for fast dehydration. Salt stress treatment was achieved by drenching 150 mM NaCl (Sinopharm Chemical Reagents Co., Shanghai, China) solution to the rice seedlings. For cold stress, seedlings were transferred to a growth chamber at 4 °C. Heat stress was applied by transferring the seedlings to a chamber with temperature at 42 °C. Leaf samples were harvested for all four abiotic stress treatments and root samples were only collected for drought and salt treatments. For hormone treatment, seedlings were sprayed with 100 µM ABA, 100 µM methyl jasmonate (MeJA) (Sigma-Aldrich, St. Louis, MO, USA), 100 µM ACC (Sigma-Aldrich, St. Louis, MO, USA), 150 µM SA (Sigma-Aldrich, St. Louis, MO, USA) in a solution containing 0.1% ethanol (Sinopharm Chemical Reagents Co., Shanghai, China) and 0.02% Tween-20 (Sinopharm Chemical Reagents Co., Shanghai, China) or with the solution as controls. All samples were stored at −80 °C until use.

### 4.3. Inoculation with Different Pathogens

*M. oryzae* strain 85-14B1 was cultivated on complete medium at 25 °C for 10 days and spores were collected to prepare inoculum. Two-week-old H8S and H8R seedlings at thee leaf stages were inoculated spraying with 5 × 10^5^ conidia/mL spore suspension containing 0.02% Tween-20 or with similar volume of solution containing only 0.02% Tween-20 as mock-inoculation controls [46]. Inoculated plants were kept under moist conditions in the dark at room temperature for 24 h and then moved to a growth chamber (12 h 28 °C light/12 h 24 °C dark). *Xoo* strain PXO99^A^ was grown in NA broth at 28 °C with shaking and cells were collected by centrifugation and diluted in distilled water to OD_600_ = 0.8. Eight-week-old IR-BB10 and IR24 plants were inoculated using leaf clipping method [73]. *R. solani* strain GD-118 (provide by Dr. Weiliang Chen, Zhejiang University, Hangzhou, China) was grown in 250 mL of potato dextrose broth medium and incubated on a 28 °C shaker for 3 days and the culture was centrifuged to collect the mycelia. Six-week-old cv. Yuanfengzao plants were inoculated by closely attaching mycelial balls (8 mm in diameter) on the sheath with aluminium foil [74]. Mock-inoculation control plants were only inoculated by attaching aluminium foil onto sheath without mycelial balls. Samples were collected from pathogen-inoculated and mock-inoculated plants at indicated time points and stored at –80 °C until use.

### 4.4. qRT-PCR Analysis of Gene Expression

Total RNAs were extracted from frozen samples using TRIzol reagent (Invitrogen, Shanghai, China) and then treated with RNase-free DNase (TaKaRa, Dalian, China). First-strand cDNA was synthesized from 1 μg total RNA using AMV reserves transcriptase (TaKaRa, Dalian, China) according to the manufacturer’s instructions. According to the previously suggested guidelines [40,75], thee different reference genes were used to assess the accuracy of qRT-PCR data and no template controls were always included in qRT-PCR experiments. Each qRT-PCR reaction contained 12.5 µL of SYBR premix Ex Taq (TaKaRa, Dalian, China), 1 µL of cDNA samples and 10 µM gene-specific primers (Table 2) in a final volume of 25 µL and was performed on a CFX96 Real-time System (Bio-Rad, Hercules, CA, USA). The relative gene expression data were calculated using the 2^−ΔΔ*C*t^ method. Rice *Actin* (accession number KC140129), *GAPDH* (accession number AK062215) and *Ubiquitin* (accession number AK059011) genes were used as internal reference controls. Thee independent biological replicates were conducted for all experiments.

**Table 2:** Primers used in this study.

Primers	Sequences (5′-3′)	Size (bp)
**Cloning of cDNAs**		
OsMC1-F	ATGGATCACTTCGGCGGACG	1107
OsMC1-R	TTACAGGACGAACGGCTTGC	
OsMC2-F	ATGGCGAGCGCGAGGCCGCC	1110
OsMC2-R	TCACAAGAGGAAGGGCTTCC	
OsMC3-F	ATGGGCTGCAACTGCCTCGT	1203
OsMC3-R	TCACAGGAGAAACGGTTTCC	
OsMC4-F	ATGGGGCGGAAGAGAGCGGT	1230
OsMC4-R	TTAGCATATGAAAGCCACGT	
OsMC5-F	ATGGGGGGCCGGAAGCGCGC	1263
OsMC5-R	TCAGCATATGAAGGCCACAC	
OsMC6-F	ATGGGCCGCAAGCGCGCGCT	1254
OsMC6-R	TCAGCATATAAAAGACACAT	
OsMC7-F	ATGGAGAGGGGTCAGAAGAA	1026
OsMC7-R	TCAGAGCGCCGTCATGGCCT	
OsMC8-F	ATGGCGGTCGTCAGCGGCGG	909
OsMC8-R	TCACAGGATAAACTGCTCCT	
OsLSD1-F	ATGTGCATTGCTGAACCAAT	606
OsLSD1-R	TCAGCTGCTGGGCTTCTGGT	
OsLSD2-F	ATGGTGGCTTCAAGAGCTCCA	444
OsLSD2-R	CTATCCTAGACTGAAAAGCA	
OsLSD3-F	ATGCAGAGCCAGATCGTGT	519
OsLSD3-R	TTACTTTTTACCACCAGTTGTA	
OsLSD4-F	ATGCAGAGCCAGATCGTGTG	561
OsLSD4-R	CTATTTCCCAGTTGTAACTCCA	
OsLSD5-F	ATGCAGGACCAGCTGATCTG	444
OsLSD5-R	TCATCTTTTCCATGAGGTGAC	
**Subcellular Localization Assays**		
OsMC1-GFP-F	CGCGGATCC ATGGATCACTTCGGCGGACG	1107
OsMC1-GFP-R	TGCTCTAGA TTACAGGACGAACGGCTTGC	
OsMC2-GFP-F	CGCGGATCC ATGGCGAGCGCGAGGCCGCC	1110
OsMC2-GFP-R	TGCTCTAGA TCACAAGAGGAAGGGCTTCC	
OsMC3-GFP-F	CGCGGATCC ATGGGCTGCAACTGCCTCGT	1203
OsMC3-GFP-R	TGCTCTAGA TCACAGGAGAAACGGTTTCC	
OsMC4-GFP-F	CGCGGATCC ATGGGGCGGAAGAGAGCGGT	1230
OsMC4-GFP-R	TGCTCTAGA TTAGCATATGAAAGCCACGT	
OsMC5-GFP-F	CGCGGATCC ATGGGGGGCCGGAAGCGCGC	1263
OsMC5-GFP-R	TGCTCTAGA TCAGCATATGAAGGCCACAC	
OsMC6-GFP-F	CGCGGATCC ATGGGCCGCAAGCGCGCGCT	1254
OsMC6-GFP-R	TGCTCTAGA TCAGCATATAAAAGACACAT	
OsMC7-GFP-F	CGCGGATCC ATGGAGAGGGGTCAGAAGAA	1026
OsMC7-GFP-R	TGCTCTAGA TCAGAGCGCCGTCATGGCCT	
OsMC8-GFP-F	CGCGGATCC ATGGCGGTCGTCAGCGGCGG	909
OsMC8-GFP-R	TCCCCCGGG TCACAGGATAAACTGCTCCT	
**qRT-PCR Assays**		
OsMC1-RT-F	GCTTCATCAAGGCGGTGGAGTG	142
OsMC1-RT-R	AAGTTGGCGACCTTGCGGATG	
OsMC2-RT-F	CGACCCGTACAGGGTGCCGA	166
OsMC2-RT-R	GCACAGCGCCTCGTCGTAGC	
OsMC3-RT-F	GGCTCCTTCGTCCGCAAGAT	101
OsMC3-RT-R	CACAGGAGAAACGGTTTCCTGT	
OsMC4-RT-F	TCGACGTTCGTGGAGATGCTC	126
OsMC4-RT-R	ATTCACGAGCCGCCTGATCTT	
OsMC5-RT-F	GTGCCAGACCGACCAGACAT	102
OsMC5-RT-R	CCGCTCTTCTCCGACAGGAT	
OsMC1-RT-F	GCTTCATCAAGGCGGTGGAGTG	142
OsMC1-RT-R	AAGTTGGCGACCTTGCGGATG	
OsMC6-RT-F	CCACACCGCAGGGTTCTTCAT	147
OsMC6-RT-R	GTCCAGGCTGCTGAGTGTATCC	
OsMC7-RT-F	ATACAGACCGTGCTGGCGTC	143
OsMC7-RT-R	AGGAATGGCGTCTCGGCGTT	
OsMC8-RT-F	TCCGGCAAGTGCCTCGTAAC	150
OsMC8-RT-R	CAATGCGGTCGGTCACAGGAT	
**Yeast Two-Hybrid Assays**		
OsMC1-BD-F	CGGAATTC ATGGATCACTTCGGCGGACGT	1107
OsMC1-BD-R	CGCGGATCC TTACAGGACGAACGGCTTGCG	
OsMC2-BD-F	CCGGAATTC ATGGCGAGCGCGAGGCCG	1110
OsMC2-BD-R	CGCGGATCC TCACAAGAGGAAGGGCTTC	
OsMC3-BD-F	CCGGAATTC ATGGGCTGCAACTGCCTCGTC	1203
OsMC3-BD-R	CGCGGATCC TCACAGGAGAAACGGTTTCC	
OsMC1-BDN-R	TGCTCTAGA CTTGCCGCGGGAGCCCGGGA	252
OsMC1-BDC-F	CGCGGATCC AAGCGCGCCGTCCTGATCGGC	855
OsLSD1-AD-F	CCGGAATTC ATGTGCATTGCTGAACCAAT	606
OsLSD1-AD-R	CGCGGATCC TCAGCTGCTGGGCTTCTGGT	
OsLSD2-AD-F	CCGGAATTC ATGGTGGCTTCAAGAGCTCCA	444
OsLSD2-AD-R	CGCGGATCC CTATCCTAGACTGAAAAGCA	
OsLSD3-AD-F	CCGGAATTC ATGCAGAGCCAGATCGTGT	519
OsLSD3-AD-R	CGCGGATCC TTACTTTTTACCACCAGTTGTA	
OsLSD4-AD-F	CCGGAATTC ATGCAGAGCCAGATCGTGTG	561
OsLSD4-AD-R	CGCGGATCC CTATTTCCCAGTTGTAACTCCA	
OsLSD5-AD-F	CCGGAATTC ATGCAGGACCAGCTGATCTG	444
OsLSD5-AD-R	CGCGGATCC TCATCTTTTCCATGAGGTGAC	

### 4.5. Subcellular Localization of the OsMC Proteins

The coding sequences of the *OsMC* genes was amplified using gene-specific primers (Table 2) and cloned into pMD-19T (Takara, Dalian, China) by T/A cloning, yielding plasmids pMD-19T-OsMC1/2/3/4/5/6/7/8. After confirmation by sequencing, the coding sequences were amplified from plasmids pMD-19T-OsMC1/2/3/4/5/6/7/8 with gene-specific primers containing restriction enzyme sites and cloned into pFGC-Egfp at the corresponding restriction enzyme sites, yielding pFGC-OsMC1/2/3/4/5/6/7/8. The recombinant plasmids pFGC-OsMC1/2/3/4/5/6/7/8 and the pFGC-Egfp empty vector were transformed into *Agrobacterium tumefacies* strain GV3101 by electroporation using GENE PULSER II Electroporation System (Bio-Rad Laboratories, Hercules, CA, USA). Agrobacteria harboring pFGC-OsMC1/2/3/4/5/6/7/8 and pFGC-Egfp were grown in YEP medium (50 µg/mL rifampicin, 50 µg/mL kanamycin and 25 µg/mL gentamicin) for 24 h with continuous shaking at 28 °C, collected by centrifugation and resuspended in infiltration buffer (10 mM MgCl_2_, 10 mM MES, 200 µM acetosyringone, pH 5.7). Agrobacteria carrying pFGC-OsMC1/2/3/4/5/6/7/8 or pFGC-Egfp empty vector were infiltrated into leaves of 4-week-old *N. benthamiana* plants expressing a red nuclear marker RFP-Histone 2B protein [56] using 1 mL needless syringes and the agroinfiltrated plants were grown in a growth room at 25 °C for 48 h. Fluorescence signals were excited at 488 nm and detected using a 500–530 nm emission filter preformed with Zeiss LSM 780 confocal laser scanning microscope (Carl Zeiss, Jena, Germany).

### 4.6. Yeast Two-Hybrid Interaction Assays

Protein-protein interactions between OsMCs and OsLSDs were examined though yeast two-hybrid assay using Matchmaker Gold Yeast Two-Hybrid System according to the manufacturer’s instructions. The coding sequences of OsMC1/2/3 were amplified using gene-specific primers (Table 2) from pMD-19T-OsMC1/2/3 and inserted into pGBKT7, yielding pGBKT7-OsMC1/2/3 bait vectors or inserted into pGADT7, yielding pGADT7-OsMC1/2/3 prey vectors. The sequences for the N-terminal (1–84 aa) and C-terminal (85–368 aa) regions of the *OsMC1* gene were also amplified from pMD-19T-OsMC1 with gene-specific primers (Table 2) and cloned into pGBKT7, yielding pGBKT7-OsMC1N and pGBKT7-OsMC1C vectors. The coding sequences of the *OsLSD* genes was amplified using gene-specific primers (Table 2) and cloned into pM D-19T (Takara, Dalian, China), yielding plasmids pMD-19T-OsLSD1/2/3 and pMD-19T-OsLOL1/2. After confirmation by sequencing, the coding sequences were amplified from plasmids pMD-19T-OsLSD1/2/3 and pMD-19T-OsLOL1/2 with gene-specific primers containing restriction enzyme sites and cloned into pGADT7, yielding pGADT7-OsLSD1/2/3 and pGADT7-OsLOL1/2 prey vectors. The bait and prey vectors in different combinations were co-transformed into Y2HGold yeast cells and confirmed by colony PCR. The transformed yeasts were cultivated on SD/Trp^−^Leu^−^His^−^Ade^−^ medium for 3 days at 30 °C and added with X-α-gal (5-bromo-4chloro-3-indolyl-a-d-galactopyranoside) solution to examine the activity of β-galactosidase. Interactions were evaluated based on the growth situation of the transformed yeast cells on the SD/Trp^−^Leu^−^His^−^Ade^−^ medium and the production of blue pigment after the addition of X-α-Gal. Yeasts co-transformed with pGBKT7-53 or pGBKT7-Lam and pGADT7-T were as positive and negative controls, respectively.

### 4.7. Bioinformatics Analysis of Cis-Element in the Promoters of the OsMC Genes

Approximately 1000 bp sequences upstream of the transcriptional start site of each *OsMC* genewere downloaded from the rice genome sequence database at the Rice Genome Annotation Project (http://rice.plantbiology.msu.edu). Putative *cis*-elements in the promoter regions were identified by searching against the PLACE *cis*-element database at http://www.dna.affrc.go.jp/PLACE/signalscan.html.

### 4.8. Accession Numbers

Sequences of the *OsMC* genes used in this study were obtained from the Rice Genome Annotation Database (http://rice.plantbiology.msu.edu/) under the following identifiers: *OsMC1* (LOC_Os03g27120), OsMC2 (LOC_Os03g27210), *OsMC3* (LOC_Os03g27170), *OsMC4* (LOC_Os05g41660), *OsMC5* (LOC_Os05g41670), *OsMC6* (LOC_Os01g58580), *OsMC7* (LOC_Os11g04010), *OsMC8* (LOC_Os03g27190), *OsLOL1* (LOC_Os08g06280), *OsLOL2* (LOC_Os07g28910), *OsLSD1* (LOC_Os12g41700), *OsLSD2* (LOC_Os03g43840) and *OsLSD3* (LOC_Os08g03610).

## 5. Conclusions

As the first step toward understanding of the functions of *OsMCs*, the present study was focused on the possible involvement of *OsMCs* in abiotic and biotic stress responses though comprehensive analysis of the expression changes in rice plants treated with different abiotic stresses, three different pathogens and four well-known stress-related hormones. Our data indicate that members of the *OsMC* family respond differentially to multiple abiotic and biotic stresses as well as to stress-related hormones, as summarized in Table 3. Based on the expression patterns, *OsMC1*, *OsMC7*, and *OsMC8* may have functions in interactions with *M. oryzae*, *Xoo*, and *R. solani*, while *OsMC3*, *OsMC5*, and *OsMC6* seem to be involved in interaction with fungal pathogens such as *M. oryzae* and *R. solani*. *OsMC4* responds in compatible interactions with *M. oryzae* and *Xoo* while *OsMC2* specifically responds in incompatible rice-*Xoo* interaction. *OsMC4*, *OsMC6*, and *OsMC7* in root tissues respond with similar patterns under drought and salt stresses. *OsMC2* is specifically downregulated by drought stress while *OsMC5* and *OsMC8* are specifically downregulated by salt stress. *OsMC1* showed opposite expression patterns in response to drought and salt stresses, whereas *OsMC3* did not show expression change in these two stresses. *OsMC3*, *OsMC4*, and *OsMC5* displayed opposite expression patterns in response to heat and cold stresses, respectively, while expression of *OsMC7* is downregulated by both heat and cold stresses. *OsMC1* and *OsMC6* are upregulated by heat stress but not by cold stress. Importantly, our data revealed that the OsMC proteins have different subcellular localizations and type I metacaspases OsMC1 and OsMC3 can interact with OsLSD1 or OsLSD3. Results from this systematic analysis of the *OsMC* family not only provide evidence for the possible involvement of *OsMCs* in response to abiotic and biotic stresses, but also provide clues for further functional analysis of *OsMCs* genes in stress tolerance and pathogen resistance in rice.

**Table 3:** Summary on the expression patterns of OsMC*s* in response to pathogens, abiotic stresses and hormones.

Genes	*M. oryzae*		*Xoo*		*R. solani*	Abiotic Stress				Hormones			
	Compatible	Incompatible	Compatible	Incompatible		Drought	Salt	Heat	Cold	ACC	JA	SA	ABA
*OsMC1*	↓	↑	↓	--	↑	↓	↑	↑	--	↑	↓	↑	↑
*OsMC2*	--	--	--	↑	--	↓	--	--	--	↓	--	↓	↑
*OsMC3*	↓	↑	--	--	↓	--	--	↑	↓	--	--	--	--
*OsMC4*	↓	--	↑	--	--	↓	↓	↓	↑	↓	--	↑	↓
*OsMC5*	↑	↑	--	--	↓	--	↓	↑	↓	--	--	--	↓
*OsMC6*	--	↑	--	--	↓	↓	↓	↑	--	↑	--	--	↓
*OsMC7*	↑	↑	↓	--	↑	↑	↑	↓	↓	--	↓	↓	↑
*OsMC8*	↓	--	--	↑	↓	--	↓	--	--	--	--	↓	--

↑, represents significant upregulation; ↓, indicates significant downregulation; -- indicates no significant change. Expression patterns in root under drought and salt stresses are listed.
